# The temporal trends of incidence, prevalence, mortality, and disability-adjusted life years of inflammatory bowel disease in the Chinese population aged 0–54 years and prediction to 2030

**DOI:** 10.3389/fmed.2026.1689227

**Published:** 2026-02-25

**Authors:** Xin Jiang, Na Xie, Yaqin Hu

**Affiliations:** 1 Chongqing General Hospital, Chongqing, China; 2 Chongqing Medical University, Chongqing, China

**Keywords:** age-period-cohort, disease burden, inflammatory bowel disease, Joinpoint, prediction, trends

## Abstract

**Background:**

The Global Burden of Disease (GBD) Study 2021 highlights that the age-standardized incidence rate of inflammatory bowel disease (IBD) in China reaches its peak prior to the age of 55 years, comprehensive analyses of the temporal trends in IBD burden among populations aged 0–54 years remain limited. To address this gap, we quantified the epidemiological trends of IBD in this age stratum from 1992 to 2021 and projected future trends through 2030.

**Methods:**

Age-standardized incidence (ASIR), prevalence (ASPR), mortality (ASMR) and disability adjusted life years (ASDR) rates for all ages attributable to IBD, together with the corresponding absolute numbers, were extracted from the GBD 2021database for the period 1992–2021. All metrics were stratified by gender and 5-year age groups. The ASIR, ASPR, ASMR, ASDR were re-estimated using the World Health Organization’s standard population. Temporal trends were analyzed using Joinpoint regression to calculate average annual percentage changes (AAPCs), and an age-period-cohort model was utilized to elucidate the distinct influences of age, period and birth cohort on IBD burden. Finally, autoregressive integrated moving average (ARIMA) models were constructed to forecast ASIR, ASPR, ASMR and ASDR rates for the population aged 0–54 years through 2030.

**Results:**

From 1992 to 2021, IBD affected 227,772 new cases, with 1,530,231 prevalent cases and 17,291 deaths, resulting in a total of 1,264,376 disability-adjusted life years (DALYs) among populations aged 0–54 years. The ASIR exhibited a gradual increase in both males (AAPC = 2.23, 95%CI: 2.09–2.34%, *p* < 0.001) and females (AAPC = 2.24, 95%CI: 2.09–2.36%, *p* < 0.001). Similarly, the ASPR also demonstrated an upward trend for males (AAPC = 1.65, 95%CI: 1.52–1.77%, *p* < 0.001) and females (AAPC = 1.55, 95%CI: 1.40–1.67%, *p* < 0.001). Conversely, the ASMR and ASDR significantly decreased in both males and females during the same period. The AAPC for ASMR was −3.41% for males (95%CI, −3.67 to −3.21%, *p* < 0.001) and −5.94% for females (95%CI, −6.10 to −5.77%, *p* < 0.001). For ASDR, the AAPC was −2.95% for males (95%CI, −3.09 to −2.80%, *p* < 0.001) and −4.72% for females (95%CI, −4.86 to −4.58%, *p* < 0.001). The effects of age, period, and cohort on the incidence, prevalence, mortality, and DALYs associated with IBD varied significantly. Projections indicate that by 2030, the ASIR and ASPR are projected to rise, whereas the ASMR and ASDR are expected to decline.

**Conclusion:**

The burden of IBD in China is substantial and continues to intensify among populations aged 0–54 years. To mitigate this growing medical burden, it is imperative to establish a comprehensive three-tiered prevention strategy, promote effective health education, implement early screening programs, ensure timely diagnosis and treatment, and improve the quality of life of individuals living with IBD.

## Introduction

Inflammatory bowel disease (IBD) is a kind of multifactorial condition, aberrant, immune-mediated, chronic, and recurrent inflammatory condition of the gastrointestinal tract and can persist and relapse throughout the patients’ lifetime (1). IBD is primarily characterized by ulcerative colitis (UC) and crohn’s disease (CD). Its etiology and pathogenesis of IBD are complex, which involves genetic, environmental, psychological, immunologic factors and alterations in the gut microbiota (2). The IBD typically presents with abdominal pain, diarrhea, fever, intestinal obstruction, anemia, and elevated inflammatory markers. Persistent intestinal inflammation increases the risk of both gastrointestinal and extraintestinal malignancies, particularly colorectal cancer (3). Patients with IBD have a significantly elevated risk of developing colorectal cancer compared to the general population, with cumulative incidences of approximately 2, 8, and 18% at 10, 20, and 30 years after diagnosis, respectively (4). Consequently, IBD progressively consumes disproportionate amount of medical resources and imposes incrementally management challenges.

The epidemiological landscape of IBD has shifted markedly, with a gradual stabilization of the incidence rate in high-income countries (5), whereas a pronounced upturn in newly industrialized regions across South America, Eastern Europe, Asia and Africa, including mainland China (6, 7). The rapid growth of the global economy and industrialization had driven a widespread shift toward westernized lifestyles among the Chinese population, including unhealthy dietary pattern (e.g., high intake of red or processed meat, low consumption of fruits and vegetables), increased sedentary behavior, reduced physical activity, and substance abuse (e.g., alcohol and tobacco), all of which were strongly implicated in the rising incidence of IBD observed in China (8). IBD is a lifelong disorder that requires continuous pharmacological and multidisciplinary care (9). Persistent intestinal inflammation impairs digestive function and significantly diminishes health related quality of life. Recurrent abdominal pain, frequent need for hospitalization, repeated endoscopic procedures, and the risk of possible complications such as colorectal cancer imposed substantial physical, psychological, and socioeconomic burdens. Indeed, 30.6% of patients with IBD allocated more than 50% of their annual household income to direct medical expenses (10). These observations underscore the rapidly escalating burden of IBD in China, which is driven by socio-economic development and lifestyle changes. This necessitates a comparative analysis of disease burden in China to enable better healthcare resource allocation and planning.

IBD has evolved into a truly global disease, with an estimated 375,140 cases worldwide and 1,510,784 per 100,000 person-year due to disability-adjusted life years in 2021 (11). The prevalence of IBD in East Asia is projected to increase 1.5 fold by 2035 compared to 2020, reaching an estimated 4.5 million cases (12). In 2021, the prevalent cases of IBD in China was estimated to be 168,077, with 24,941 new cases and 5,640 deaths, resulting in 136,932 total DALYs (13). Accurate characterization of the current epidemiological trajectory of IBD in China is essential for developing evidence-based control, management, and prevention strategies, ultimately resulting in substantial savings in both healthcare resources and government expenditures. Previous study in China had predominantly quantified disease burden across the entire age continuum (13) and demonstrated that the incidence of IBD reached its peak before 55 years. The rising incidence of IBD in early adulthood has increasingly focused attention on this demographic, highlighting the need for rigorously targeted studies. Given that IBD is most prevalent among young adults, and is characterized by a prolonged clinical course with recurrent inflammatory flares (14), understanding its epidemiology in this age group is crucial for effective management and prevention. Additionally, approximately 25% of IBD cases have their onset during childhood (15), the disease burden of IBD in children and younger adolescents is often under recognized and widespread (16). Consequently, rigorous population-based epidemiological studies are urgently needed, especially among populations aged 0–54 years to redress the current evidence deficit and accurately quantify the burden of IBD in China.

Due to the absence of a nationwide registry, nationally representative, population-based epidemiological data on IBD in China remain scarce. Only a few studies had focused on the incidence or prevalence in limited regions (17). In addition, epidemiological studies of IBD in China have primarily focused on descriptive statistics, with no systematic prediction of its future development (18, 19). Marked heterogeneity in IBD burden estimates for China had emerged across the 2017 and 2019 iterations of the GBD study, primarily due to successive refinements in modeling algorithms and data-processing pipelines. Such discrepancies introduce significant uncertainty when quantifying the true epidemiological profile of the Chinese population aged 0–54 years. Therefore, the establishment of an updated database is urgently needed to generate robust, detailed estimates that can reliably inform evidence-based health policy and resource allocation. Timely and systematic updates of epidemiological data on IBD in China are essential to delineate its contemporary clinical and demographic profile and to provide an evidence-based framework for the rational allocation of public-health resources.

While previous studies based on GBD data have delineated the overall national epidemiological patterns of IBD, systematic evidence remains scarce regarding the long-term, fine-grained evolution of disease burden among individuals aged 0–54 years in China. This age group bears the highest disease burden and exerts the greatest impact on social productivity. In particular, comprehensive analyses disentangling the respective contributions of age, period, and birth cohort effects are lacking, as are quantitative projections of future disease burden trends within this critical population. In this study, we compiled the latest estimates from the GBD study in 2021 to evaluate trends in IBD among the populations aged 0–54 years by gender and age, and to analysis the effect of period, cohort effect using GBD data in China over the past 30 years. Additionally, we conducted short-term predictions for the disease burden, which are indispensable for formulating evidence based on disease control policies and optimizing the allocation of healthcare services. Moreover, this study contextualized its findings within the framework of China’s profound historical and socioeconomic transformations, thereby addressing growing demand for higher quality health and well-being among the Chinese population.

## Methods

### Data source

The data utilized in this study were obtained from GBD 2021 database. The GBD study employs rigorously standardized and replicable modeling tools—most notably the Bayesian meta-regression platform DisMod-MR 2.1 and spatiotemporal Gaussian process regression to synthesize global epidemiological evidence from diverse sources, including published studies, hospital records, and surveillance registries. Through systematic data integration and the imputation of missing values, this methodological framework generates continuous and comparable time-series estimates at both national and global levels. By explicitly quantifying uncertainty and minimizing dependence on sparse or incomplete national surveillance systems, the GBD approach produces robust composite indicators even in data-scarce settings, thereby ensuring the temporal and cross-jurisdictional comparability essential for policy translation.

Specifically, we extracted data on the number of incident cases, prevalent cases, deaths, and disability-adjusted life years (DALYs) related to IBD in China, along with the corresponding age-standardized rates (ASR) (per 100,000 population), including age-standardized incidence rate (ASIR) age-standardized prevalence rate (ASPR), age-standardized mortality rate (ASMR), and age-standardized DALY rate (ASDR), along with their respective 95% uncertain interval (UI), covering the period from 1992 to 2021. Data were stratified by gender, age group, and years using the GBD Results tool. In this study, we focused on the populations aged 0–54 years based on a previous study (13), which identified a peak incidence rate of IBD among populations aged 50–54 years, with a subsequent decline in incidence in subsequent age groups. The age of 50–54 years was considered an inflection point in the incidence of IBD. To facilitate detailed analysis, the population aged 0–54 years was divided into 11 age intervals: “0–4,” “5–9,” “10–14,” “15–19,” “20–24,” “25–29,” “30–34,” “35–39,” “40–44,” “45–49,” and “50–54” years. The population data for China used in GBD2021 study were also obtained from the GBD Results tool.

### Statistical analysis

The ASIR, ASPR, ASMR, and ASDR for the populations aged 0–54 years were recalculated using the World Health Organization (WHO)’s 2000–2025 world standard population as the reference. The calculation process for ASR involved the following steps: 1. Calculate the total percentage of the population aged 0–54 years in the WHO’s world standard population. 2. Calculate the standard population weight (*β_k_*) for each age group within this range. 3. Compute the weighted incidence, prevalence, mortality and DALYs rate by multiplying the age-specific crude rates (α*_k_*) by the corresponding standard population weight. 4. Sum the weighted incidence, prevalence, mortality and DALYs rates for the different age groups to obtain the standardized rate for the 0–54 years age range. The calculation formula was as fellows:


$$\mathrm{ASR}=\frac{\sum_{k=1}^{n}\alpha_{k}\beta_{k}}{\sum_{k=1}^{n}\beta_{k}}\times 100,000$$

This procedure was applied to the age-specific crude rates of incidence, prevalence, mortality, and DALYs for each year and age group to derive the ASIR, ASPR, ASMR, and ASDR of the population aged 0–54 years.

The temporal trends of ASIR, ASPR, ASMR, and ASDR for IBD among the population aged 0–54 years from 1992–2021 by gender and age were analyzed using Joinpoint regression analysis. The annual percentage change (APC) and average annual percentage change (AAPC), along with their corresponding 95% confidence intervals (CI), were calculated using the Joinpoint Regression Program developed by the National Cancer Institute. Additionally, *t*-tests were employed to determine the significance of trend changes. In the Joinpoint regression model, the dependent variables were ASIR, ASPR, ASMR, and ASDR, with calendar year as the independent variable. The model parameters were set as follows: the heteroskedasticity/correlated error option was set to standard error (provided), and the maximum number of Joinpoints was set to five. A log-linear model of the form “ln(y) = xb” was selected, and the Bayesian information criterion (BIC) was utilized to determine the optimal number of change points. Trends in ASIR, ASPR, ASMR, and ASDR were interpreted as follows: an increasing trend was identified if both the APC/AAPC and the lower limit of the 95%CI were positive (*p* < 0.05); a decreasing trend was inferred if both the APC/AAPC and the upper limit of the 95%CI were negative (*p* < 0.05). If neither condition was satisfied, the ASIR, ASPR, ASMR, and ASDR were considered stable (20).

Age-period-cohort (APC) analysis quantifies population-level disease burden by simultaneously modeling age, period, and cohort effects via Poisson regression. We applied the publicly available APC tool^1^ to examine the age, period, and cohort specific trends in China from 1992 to 2021. To improve temporal resolution, we extracted national disease burden data among populations aged 0–54 years for the single index years 1994, 1999, 2004, 2009, 2014, and 2019. These years were used to represent the periods “1992–1996,” “1997–2001,” “2002–2006,” “2007–2011,” “2012–2016,” and “2017–2021,” respectively, based on previous studies (21–23). Participants were stratified into 11 contiguous 5-year age groups, including “0–4,” “5–9,” “10–14,” “15–19,” “20–24,” “25–29,” “30–34,” “35–39,” “40–44,” “45–49,” and “50–54” years. Because age, period, and cohort are linearly dependent (cohort = period-age), the APC model is not identifiable without additional constraints. Therefore, we applied the intrinsic estimator (IE) (24) to address this challenge. Following the web tool’s defaults, we set the 0–4 age group, 2002–2006 period, and 1977–1981 cohort as reference categories.

The 0–4 year age group was designated as the reference category, as it represents the initial demographic segment of the study population. From an epidemiological perspective, this group provides an intuitive baseline for assessing relative risks across other age groups, given that IBD incidence in early childhood is typically minimal.

The period 2002–2006 was selected as the reference period. During the early 2000s, China’s healthcare system, particularly the availability and utilization of diagnostic technologies such as digestive endoscopy were expanded rapidly in medium and large cities. Consequently, the interval from 2002 to 2006 can be regarded as a phase in which IBD diagnostic capacity reached a relatively stable and broadly improved level, rendering it an appropriate reference for period comparisons.

The birth cohort of 1977–1981 was chosen as the reference cohort because its members entered early adulthood (approximately 20–30 years of age) around the turn of the millennium, a critical life stage during which IBD risk begins to increase markedly. Using this cohort as the baseline enabled a clearer evaluation of relative risk differences among cohorts born before and after this period.

The APC model produces three primary parameters: (1) Net drift: the overall annual log-linear change in ASR across the entire study period. (2) Longitudinal age curve: age-specific rate ratios after full adjustment for period and cohort effects, describing risk as a smooth function of age. (3) Rate ratio (RR): relative risks for a specific cohort (or period) compared to the reference cohort (or period), after adjusting for age and nonlinear period effects. It quantifies the impact of cohort (or period) on mortality risk, independent of age and other confounding factors.

The short term future trends of IBD on ASIR, ASPR, ASMR, and ASDR among population aged 0–54 years were projected using autoregressive integrated moving average (ARIMA) model, which was selected for its parsimony and proven effectiveness in public-health time series forecasting. Model performance was evaluated by root mean square error (RMSE), mean square error (MSE), mean absolute error (MAE), and mean absolute percentage error (MAPE), with smaller values indicating higher accuracy. Ultimately, only models with MAPE less than 5% were considered acceptable. Model adequacy was verified using the Ljung-Box *Q*-test; residuals were deemed white-noise (*p* > 0.05), confirming the absence of autocorrelation and validating the model’s predictive utility.

Crude incidence, prevalence, mortality, and DALYs rate and ASRs, including ASIR, ASPR, ASMR, and ASDR among population aged 0–54 years were computed in Excel 2013. Temporal trends of ASIR, ASPR, ASMR, and ASDR among population aged 0–54 years were assessed with Joinpoint regression model (version4.6.0.0, Applications Branch, National Cancer Institute, Bethesda, United States). APC model was performed via the web tool.^2^ Predictions to 2030 were conducted by R software (4.2.0). Figures were produced in GraphPad Prism 8.0. All tests were two-sided, with *α* = 0.05.

## Results

### Disease burden of IBD among the populations aged 0–54 years stratified by gender in China from 1992 to 2021

**Figure 1:**
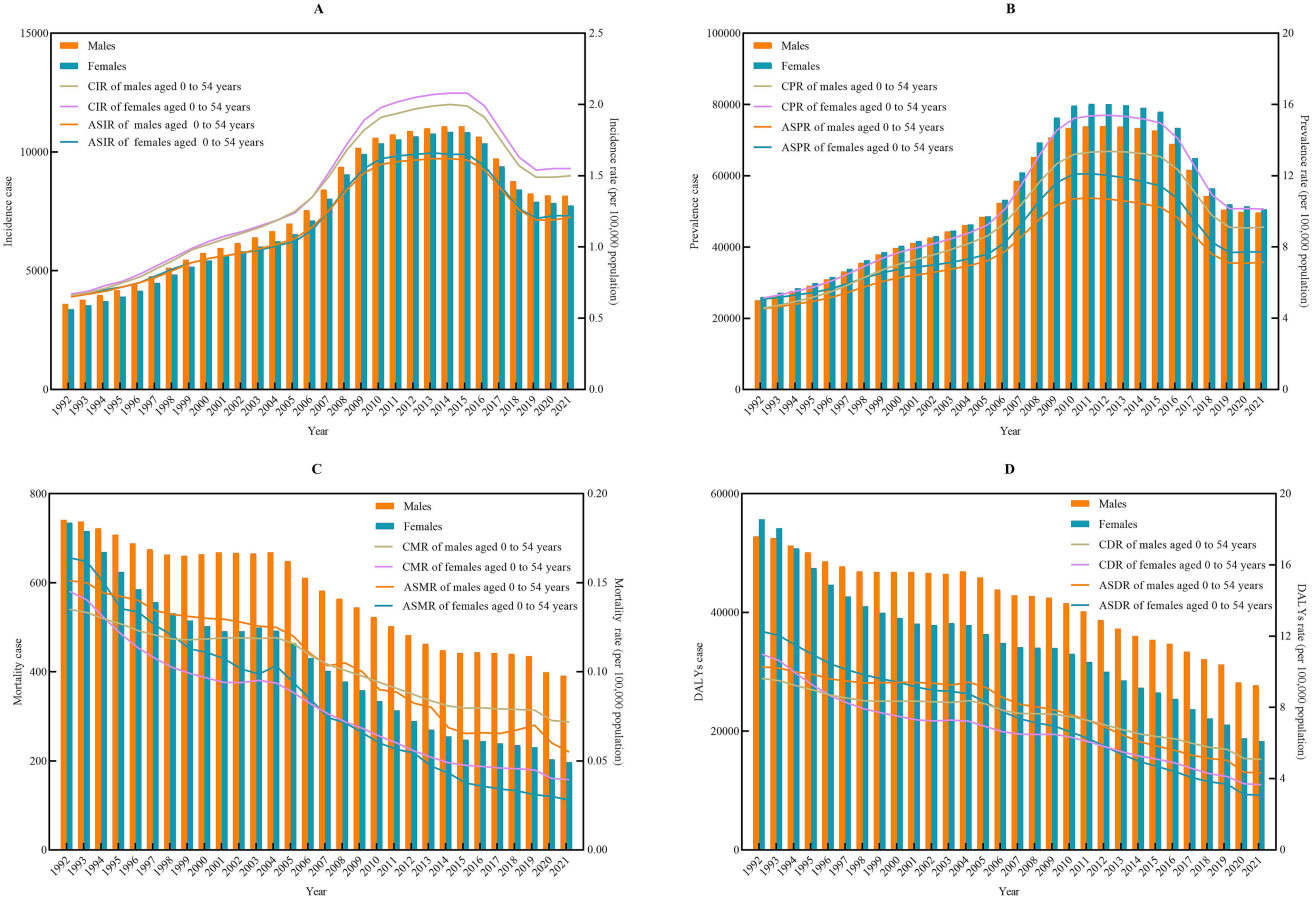
Number and sex-specific rates of incidence, prevalence, mortality, and DALYs among population aged 0–54 years due to inflammatory bowel disease in China from 1992 to 2021. **(A)** Incidence; **(B)** prevalence; **(C)** mortality; and **(D)** DALYs. CIR, crude incidence rate; CPR, crude prevalence rate; CMR, crude mortality rate; CDR, crude disability-adjusted life years rate; ASIR, age standard incidence rate; ASPR, age standard prevalence rate; ASMR, age standard mortality rate; ASDR, age standard disability-adjusted life years rate. DALYs, disability-adjusted life years.

Based on data from the GBD study spanning from 1992 to 2021, the incident cases of IBD among males aged 0–54 years in China was 227,772, representing 75.55% of the total male population. This cohort experienced 1,530,231 prevalent cases and 17,291 deaths, resulting in a total of 1,264,376 DALYs. For females aged 0–54 years, the incident cases were 218,590, accounting for 74.34% of the total female population, with 1,600,338 prevalent cases and 12,497 deaths, leading to a total of 1,047,089 DALYs. Regarding age-standardized rates, the ASIR, ASPR, ASMR, and ASDR for males were 0.65, 4.54, 0.15, and 10.28 per 100,000 population in 1992, respectively. By 2021, these rates had changed to 1.21, 7.15, 0.06, and 4.34 per 100,000 population, respectively. For females, the corresponding rates in 1992 were 0.66, 5.07, 0.16, and 12.27 per 100,000 population, respectively, and in 2021, they were 1.22, 7.73, 0.03, and 3.09 per 100,000 population, respectively (Figure 1; Supplementary Tables 1–4).

### Trends of the disease burden of IBD among the populations aged 0–54 years stratified by gender and age from 1992 to 2021 by Joinpoint regression analysis

From 1990 to 2021, the ASIR of IBD among populations aged 0–54 years gradually increased in both males and females in China. Specifically, AAPC was 2.23% for males (95%CI, 2.09–2.34%, *p* < 0.001) and 2.24% for females (95%CI, 2.09–2.36%, *p* < 0.001). Similarly, the ASPR also exhibited an upward trend, with an AAPC of 1.65% for males (95%CI, 1.52–1.77%, *p* < 0.001) and 1.55% for females (95%CI, 1.40–1.67%, *p* < 0.001). Conversely, the ASMR and ASDR significantly decreased in both males and females during the same period. The AAPC for ASMR was −3.41% for males (95%CI, −3.67 to −3.21%, *p* < 0.001) and −5.94% for females (95%CI, −6.10 to −5.77%, *p* < 0.001). For ASDR, the AAPC was −2.95% for males (95%CI, −3.09 to −2.80%, *p* < 0.001) and −4.72% for females (95%CI, −4.86 to −4.58%, *p* < 0.001). These findings are detailed in (Table 1; Supplementary Figure 1).

**Table 1:** Joinpoint regression analysis of ASIR, ASPR, ASMR, ASDR among population aged 0–54 years.

Variable	Segment	APC	95%CI	*P*-value	AAPC	95%CI	*P*-value
ASIR							
Males	1992–2000	4.63*	4.25–5.29	<0.001	2.23*	2.09–2.34	<0.001
	2000–2005	2.70*	0.93–3.44	0.010			
	2005–2009	10.27*	8.87–12.15	0.002			
	2009–2015	1.05*	0.50–1.57	0.010			
	2015–2019	−7.97*	−9.23 to −7.19	<0.001			
	2019–2021	1.17	−1.50 to 2.94	0.232			
Females	1992–2000	4.50*	4.09–5.16	<0.001	2.24*	2.09–2.36	<0.001
	2000–2005	2.24*	0.62–3.02	0.029			
	2005–2009	11.43*	10.23–12.93	<0.001			
	2009–2015	1.04*	0.46–1.61	0.008			
	2015–2019	−8.25*	−9.57 to −7.42	<0.001			
	2019–2021	1.41	−1.34 to 3.37	0.239			
ASPR							
Males	1992–2000	4.41*	4.03–5.09	<0.001	1.65*	1.52–1.77	<0.001
	2000–2005	2.58*	0.99–3.32	0.007			
	2005–2009	10.38*	9.31–12.12	0.003			
	2009–2015	−0.18	−0.71 to 0.34	0.445			
	2015–2019	−9.50*	−10.48 to −8.77	<0.001			
	2019—2021	0.91	−1.59 to 2.75	0.365			
Females	1992–2000	3.91*	3.50–4.73	<0.001	1.55*	1.40–1.67	<0.001
	2000–2005	2.06*	0.67–2.89	0.024			
	2005–2009	12.44*	11.41–13.52	0.005			
	2009–2015	−0.32	−0.85 to 0.27	0.253			
	2015–2019	−10.23*	−11.30 to −9.43	<0.001			
	2019–2021	0.92	−1.80 to 2.91	0.402			
ASMR							
Males	1992–2004	−1.59*	−2.46 to −0.83	0.031	−3.41*	−3.67 to −3.21	<0.001
	2004–2012	−4.64*	−5.23 to −0.78	0.021			
	2012–2015	−8.43*	−10.47 to −5.43	0.001			
	2015–2019	1.52*	0.15–4.84	0.037			
	2019–2021	−10.90*	−15.05 to −6.73	<0.001			
Females	1992–1999	−5.47*	−6.94 to −4.74	<0.001	−5.94*	−6.10 to −5.77	<0.001
	1999–2004	−2.24	−3.66 to 0.33	0.080			
	2004–2016	−8.13*	−8.75 to −7.76	<0.001			
	2016–2021	−4.87*	−6.08 to −2.64	0.005			
ASDR							
Males	1992–1997	−1.79*	−3.98 to −0.74	0.009	−2.95*	−3.09 to −2.80	<0.001
	1997–2004	−0.23	−3.68 to 1.60	0.750			
	2004–2010	−3.47*	−5.80 to −1.89	0.005			
	2010–2021	−4.88*	−6.24 to −4.11	<0.001			
Females	1992–1997	−4.17*	−6.67 to −3.02	<0.001	−4.72*	−4.86 to −4.58	<0.001
	1997–2004	−2.12*	−3.21 to −0.07	0.044			
	2004–2011	−4.60*	−6.09 to −3.75	<0.001			
	2011–2021	−6.84*	−7.47 to −6.42	<0.001			

*P < 0.05. ASIR, age standard incidence rate; ASPR, age standard prevalence rate; ASMR, age standard mortality rate; ASDR, age standard disability-adjusted life years rate; APC, annual percent change; AAPC, average annual percent change; CI, confidence interval; AAPC: 1992-2021.

**Figure 2:**
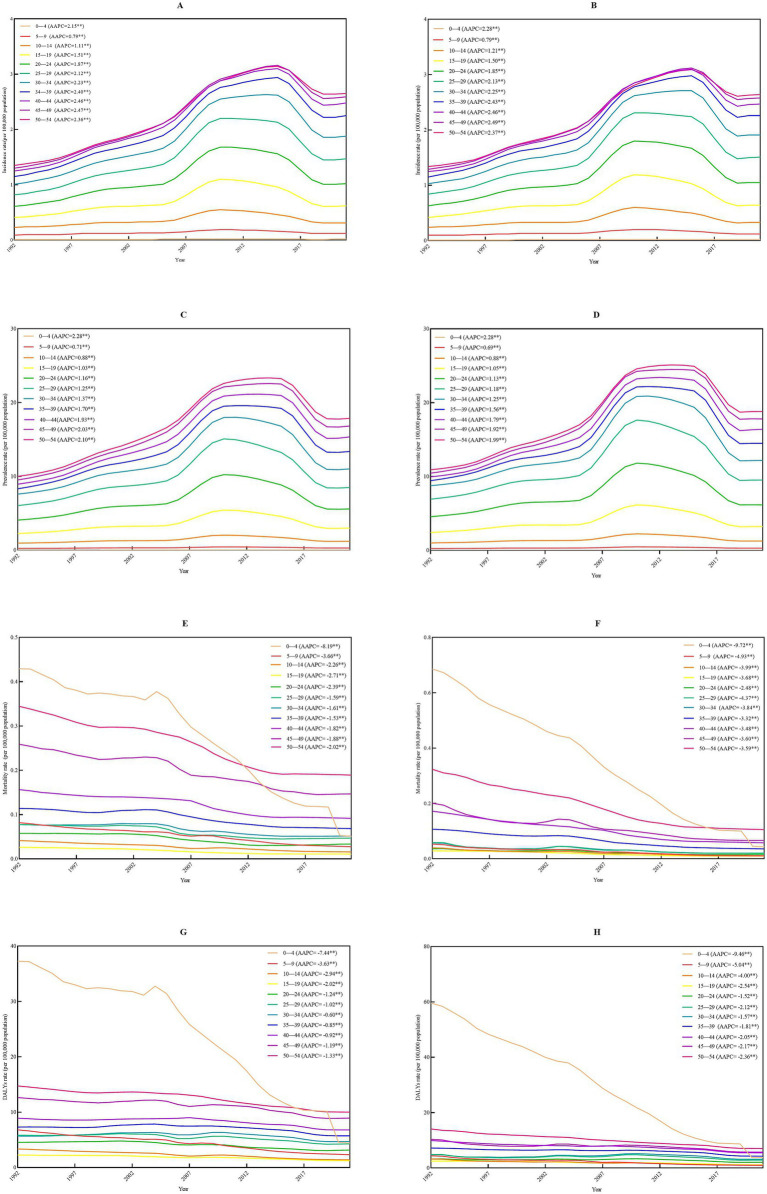
Age-specific rates of incidence, prevalence, mortality, and DALYs among population aged 0–54 years by gender due to inflammatory bowel disease in China from 1992 to 2021. **(A)** Males incidence by age; **(B)** females incidence by age; **(C)** male prevalence by age; **(D)** female prevalence by age; **(E)** male mortality by age; **(F)** female mortality by age; **(G)** male DALYs by age; **(H)** female DALYs by age. AAPC, average annual percent change; CI, confidence interval; DALYs, disability-adjusted life years.

When examining the age patterns by gender, the populations aged 45–49 years exhibited the highest AAPC among all age groups, with a rate of 2.47% (95%CI, 2.38–2.53%, *p* < 0.001) for males and 2.49% (95%CI, 2.38–2.57%, *p* < 0.001) for females from 1992 to 2021. Notably, the population aged 0–4 years exhibited the highest AAPC, with a rate of 2.28% (95%CI, 1.72–3.21%, *p* < 0.001) for males and 2.28% (95%CI, 1.75–3.25%, *p* < 0.001) for females. Despite increases in AAPC for incidence and prevalence among populations aged 0–54 years, the mortality and DALYs associated with IBD showed a statistically significant decline from 1992 to 2021. The population aged 0–4 years exhibited the greatest downward trend with an AAPC of −8.19% for males (95%CI, −9.05 to −7.21%, *p* < 0.001) and −9.72% for females (95%CI, −10.26 to −8.95%, *p* < 0.001) in mortality. For DALYs, the AAPC was −7.44% for males (95%CI, −8.24 to −6.70%, *p* < 0.001) and −9.46% for females (95%CI, −10.22 to −8.78%, *p* < 0.001). These findings were illustrated in (Figure 2; Supplementary Table 5).

### Age-period-cohort analysis of the disease burden of IBD among the populations aged 0–54 years stratified by gender from 1992 to 2021

**Figure 3:**
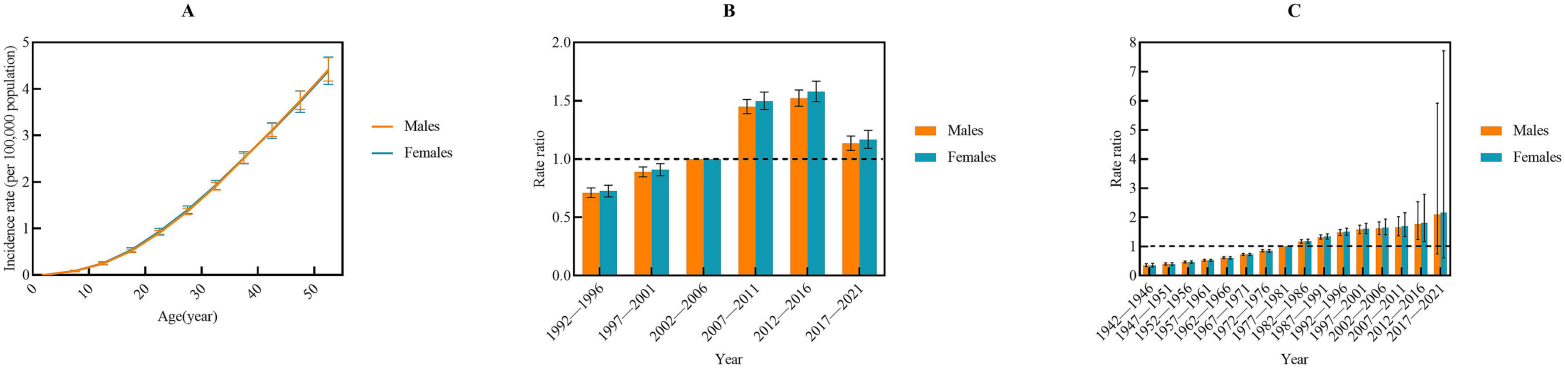
Age-period-cohort analysis of incidence rate from 1992 to 2021 by gender. **(A)** Longitudinal age curve; **(B)** period; **(C)** cohort.

The net drift in IBD incidence was 2.50 (95%CI: 2.21–2.80) for males and 2.58 (95%CI, 2.22–2.93) for females, respectively, indicating an elevated risk of IBD incidence among populations aged 0–54 years. The incidence rate increased with age, reaching a peak in the 50–54 years age group (Figure 3A). Compared with the 2002–2006 period (*RR* = 1), the relative risk of IBD incidence sharply increased sharply before declining after 2016. However, the relative risk remained above one (Figure 3B). In comparison with the 1977–1981 cohort (*RR* = 1), the relative risk of IBD incidence showed a steady increase across subsequent cohorts (Figure 3C).

**Figure 4:**
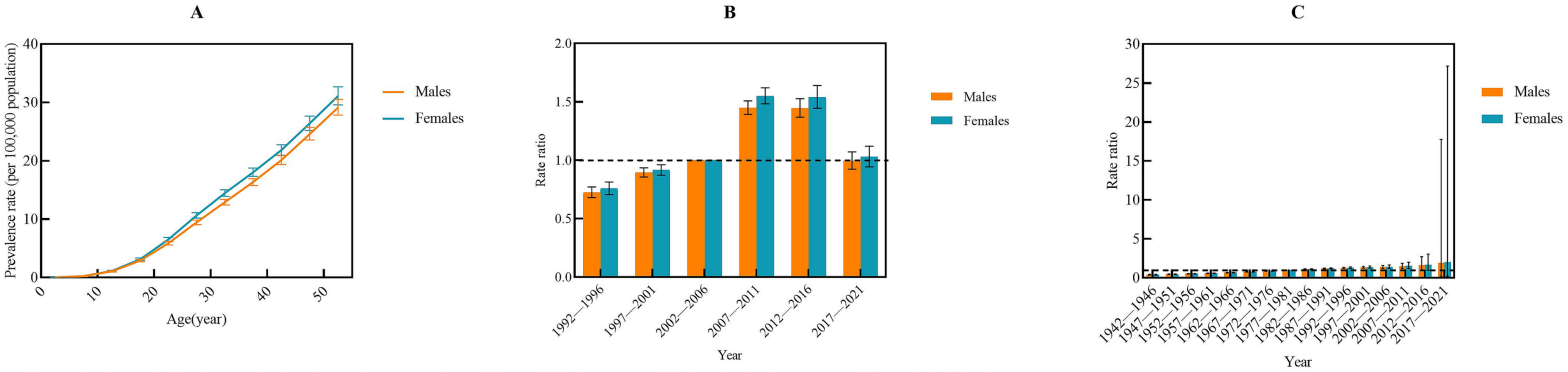
Age-period-cohort analysis of prevalence rate from 1992 to 2021 by gender. **(A)** Longitudinal age curve; **(B)** period; **(C)** cohort.

The trend in IBD prevalence mirrored that of incidence. The net drift in prevalence was 1.96 (95%CI: 1.49–2.43) for males and 2.03 (95%CI: 1.48–2.58) for females, respectively, indicating an elevated risk of IBD prevalence among populations aged 0–54 years. Similar to the incidence, the prevalence peaked in the age of 50–54 years (Figure 4A). The relative risk of IBD prevalence also exhibited a peak during the 2012–2016 period, followed by a decline in subsequent years when compared with the reference period of 2002–2006 (Figure 4B). Regarding the cohort effect, the relative risk of IBD prevalence demonstrated a similar trend to that of incidence, increasing in more recent cohorts relative to the 1977–1981 cohort (Figure 4C).

**Figure 5:**
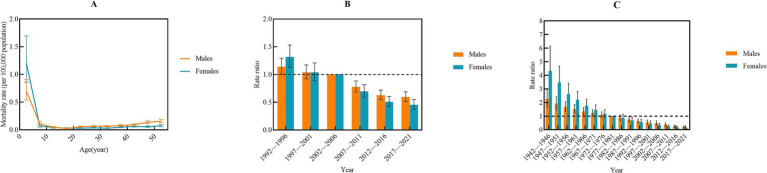
Age-period-cohort analysis of mortality rate from 1992 to 2021 by gender. **(A)** Longitudinal age curve; **(B)** period; **(C)** cohort.

The net drift of IBD mortality was −2.83 (95%CI: −3.34 to −2.30) for males and −4.38 (95%CI: −5.07 to −3.69) for females, respectively, indicating a decreased risk of IBD mortality among populations aged 0–54 years. The mortality risk decreased with increasing age (Figure 5A). However, the mortality risk was notably higher in the 0–4 age group, which is consistent with the results of the Joinpoint regression analysis conducted across different age groups. Compared with the 2002–2006 period (*RR* = 1), the relative risk of IBD mortality sharply decreased in subsequent years (Figure 5B). Additionally, the relative risk of IBD mortality declined progressively decreased over time (Figure 5C).

**Figure 6:**
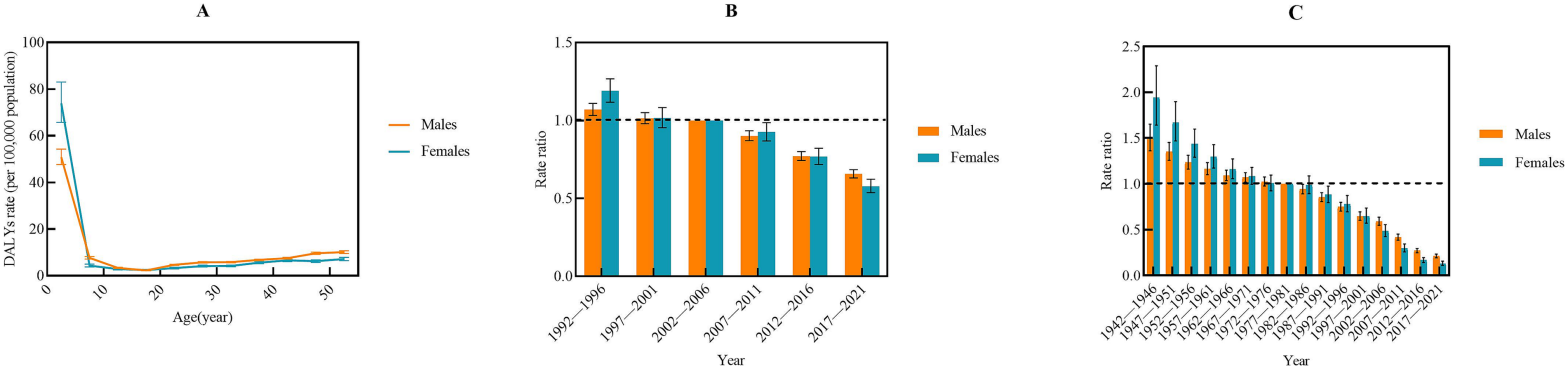
Age-period-cohort analysis of DALYs rate from 1992 to 2021 by gender. **(A)** Longitudinal age curve; **(B)** period; **(C)** cohort. DALYs, disability-adjusted life years.

The net drift of IBD-related DALYs was −1.90 (95%CI: −2.40 to −1.77) for males and −2.55 (95%CI: −2.80 to −2.30) for females, respectively, indicating a decreasing burden of IBD over time. DALYs declined with increasing age (Figure 6A). Compared with the 2002–2006 period (RR = 1), IBD-related DALYs exhibited a significant annual decline in recent years (Figure 6B). Similarly, IBD DALYs decreased progressively over time (Figure 6C).

### Prediction for the disease burden of IBD among populations aged 0–54 years stratified by gender

**Figure 7:**
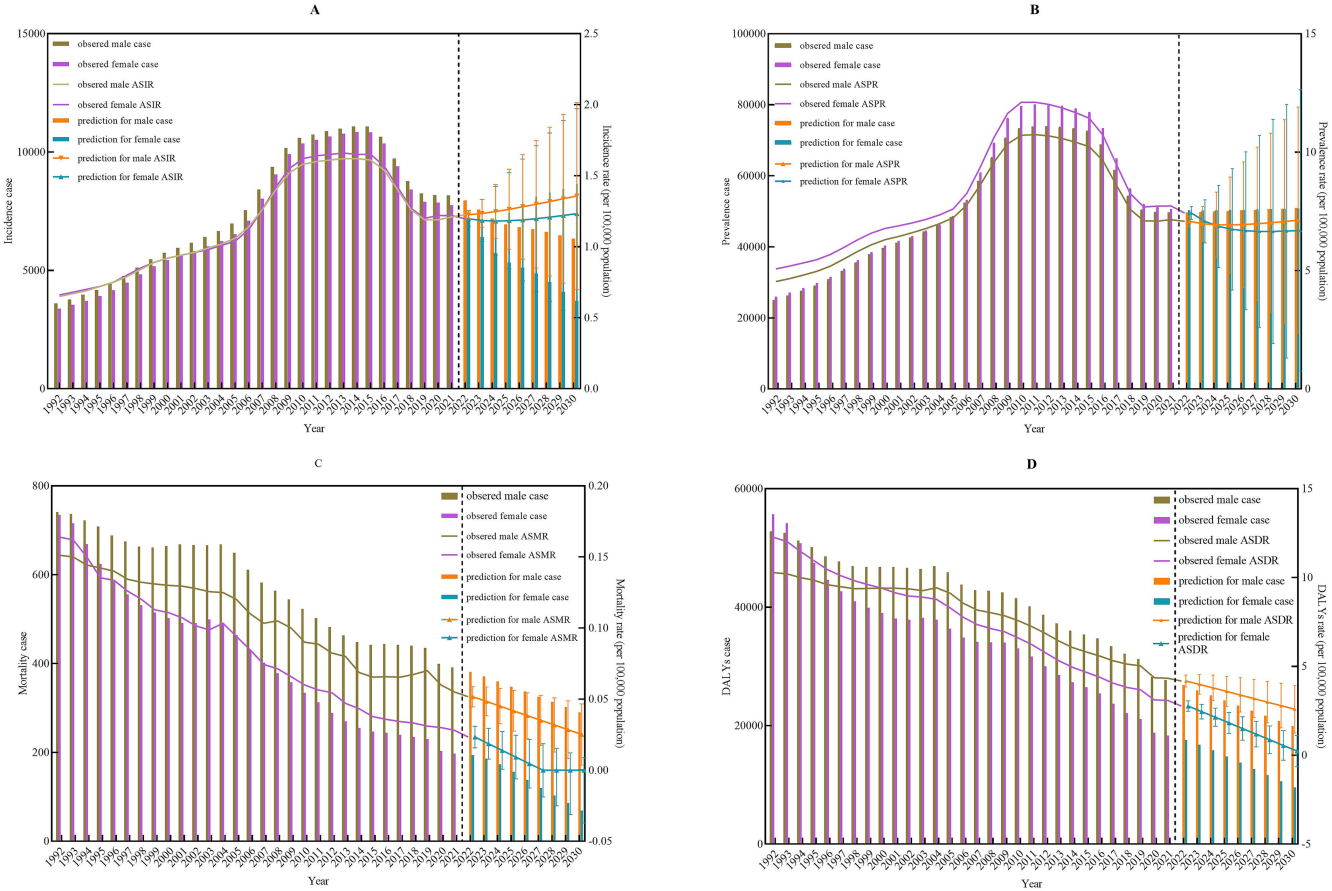
Prediction of number and sex-specific rates of ASIR, ASPR, ASMR, and ASDR among population aged 0–54 years due to inflammatory bowel disease in China **(A–D)**. ASIR, Age standard incidence rate; ASPR, Age standard prevalence rate; ASMR, Age standard mortality rate; ASDR, Age standard disability-adjusted life years rate.

By 2030, the number of incident cases of IBD is projected to reach 6,345 in males and 3,717 in females. The ASIR are expected to be 1.36 per 100,000 population in males and 1.23 per 100,000 population in females among populations aged 0–54 years, respectively. Notably, the ASIR for both males and females is anticipated to continue increasing from 2022 to 2023 (Figure 7A), with AAPC of 1.28% (95%CI: 1.25–1.33%, *P*<0.001) for males and 0.39% (95%CI: 0.28–0.53%, *P <* 0.001) for females (Supplementary Table 6). The number of prevalent cases is expected to reach 50,883 in males and 50,777 in females. The ASPR are projected to be 7.13 per 100,000 population in males and 6.69 per 100,000 population in females, respectively (Figure 7B). Throughout the projection period (2022–2030), the ASIR in males is expected to remain essentially stable (AAPC = 0.11, 95%CI: −0.03 to 0.29%; *p* = 0.112). In contrast, a statistically significant and sustained decline is projected in females (AAPC = –1.26, 95%CI: −1.49 to −0.99%; *p* < 0.001) (Supplementary Table 6). Regarding the mortality, the number of deaths is projected to be 290 in males and 69 in females. The ASMR is projected to be 0.03 per 100,000 population in males, while the ASMR in females is expected to decline to under 0.004 per 100,000 population by 2030 (Figure 7C). The ASMR is projected to decline markedly in both males and females (both *p* < 0.001). Specifically, males are projected to experience an AAPC of −8.66% (95% *CI*: −9.03 to −8.32%), whereas females are expected to exhibit an even, steeper decline, with an AAPC of −77.88% (95%CI: −91.66 to −47.90%) (Supplementary Table 6). In terms of DALYs, the projected DALYs are 19,916 person-years for males and 9,557 person-years for females by 2030. The ASDR is projected to be 2.59 per 100,000 population in males and 0.24 per 100,000 population in females, respectively (Figure 7D). The ASDR for males is expected to experience a consistent annual decline (AAPC = –5.79%; 95%CI: −5.89% to −5.69%; *p* < 0.001), while females are expected to experience an even steeper reduction (AAPC = –26.52%; 95%CI: −29.07% to −24.13%; *p* < 0.001) from 2022 to 2030 (Supplementary Table 6). The predictive performance of ARIMA model was shown in Supplementary Table 7.

## Discussion

This study delineated three decades temporal trends in the disease burden of IBD among populations aged 0–54 years in China. To our best of knowledge, it represents the first comprehensive national analysis of IBD epidemiological trajectories that integrates Joinpoint regression analysis, age–period–cohort model and ARIMA model. Qiu et al. (25) reported a straightforward yet robust analysis demonstrating a significant upward trend in both incidence and prevalence, accompanied by a concurrent decline in mortality and DALYs. Consistent findings were subsequently documented by Zhang et al. (26) and Yu et al. (13), who leveraged the GBD 2019/2021 datasets to confirm the similar trends in incidence, prevalence, and mortality rates and DALYs. Prior studies had investigated the disease burden of IBD across the entire age spectrum. However, data stratified by specific age strata remain scarce. Additionally, marked heterogeneity existed among different databases. For instance, the ASIR reported by GBD2017 was 60.37 per 100,000 population, whereas it was estimated to be 2.85 per 100,000 population in GBD2019 and 1.40 per 100,000 population in GBD2021. Benefiting from substantial methodological refinements, the GBD 2021 database provides the most accurate and up-to-date quantification of the global IBD burden.

Between 1992 and 2021, among populations aged 0–54 year, both the absolute number of incident cases and ASIR of IBD exhibited a steady increase. This secular increase can be attributable to several factors. First, rapid economic development had precipitated a marked westernization of dietary habits and lifestyle behaviors. Studies of Chinese populations had shown that increased prevalence of cigarette smoking (27), frequent consumption of deep-fried and spicy foods, excessive sugar intake (28, 29), depression, psychological stress (30), shorter duration and lower rates of breastfeeding, and widespread antibiotic exposure were associated with IBD incidence in China (31). Second, accelerating industrialization and urbanization had broadened genetic susceptibility mutations to environmental factors associated with IBD, thereby facilitating phenotypic expression and contributing to the rise in new IBD diagnoses (32). Third, the Chinese government had substantially strengthened public health policies, expanded universal healthcare coverage, and improved the accessibility and diagnostic accuracy of medical services nationwide (33). Fourth, heightened public awareness of the benefits of colonoscopic screening, combined with expanded access to endoscopic services, had led to a marked increase in screening uptake across the general population. This had uncovered previously undiagnosed or subclinical cases of IBD and contributing to the observed rise in incidence. Finally, advances in diagnostic capacity, encompassing the widespread availability of high-resolution endoscopy, cross-sectional imaging, fecal calprotectin assays, and validated serological and genetic markers, along with iterative refinement of diagnostic criteria, had markedly improved case ascertainment, resulting in the identification of a larger proportion of previously undiagnosed individuals (34).

The increase in prevalent cases and ASPR was driven by declining mortality and DALYs among populations aged 0–54 years, coupled with an increase in incident cases. These opposing trends indicated that the overall disease burden of IBD in this population had not diminished. Advances in diagnostic technology and the broader healthcare availability had enhanced physicians’ ability to diagnose, manage and prolong the survival of patients with IBD (35). The increased incidence was expected to result in a widespread and sustained prevalence of IBD. Due to its relapsing and remitting course, frequent complications, recurrent hospitalizations, surgical interventions and high treatment costs, the national disease burden of populations aged 0–54 years would be substantially amplified.

The parallel decline in ASMR and ASDR among populations aged 0–54 years aligned with the global downward trajectory (11). The overall reduction in ASMR and ASDR reflected improved survival rate among patients with IBD and was attributable to advances in therapeutic strategies, earlier diagnosis and prompt intervention (36). IBD was a chronic, relapsing, non-specific inflammatory disorder of the gastrointestinal tract. The advent of biologics, multi-cytokine-targeted combination regimens, stem cell-based interventions, and strategies that modulated the gut-brain axis and host-microbiome interactions had substantially mitigated the disease burden associated with IBD (37). Nevertheless, the substantial costs of these therapies impose a considerable financial burden on patients with IBD. Studies had shown that annual expenditure for those receiving biologics were two to three times higher than that for the average IBD patients (38).

However, the observed decline in ASMR and ASDR for IBD in China over the past three decades should be interpreted in the context of substantial advancements in both healthcare and health information systems. While genuine improvements in diagnosis and management likely contributed to this downward trend, concurrent changes in data quality and modeling may also had played a role. Key factors included the evolving accuracy, specificity, and completeness of cause-of-death attribution within China’s national mortality surveillance system, particularly regarding the coding of IBD-related deaths. Additionally, iterative updated to the GBD modeling framework may influence trend estimates across different versions. Thus, the observed decline in ASMR and ASDR should be regarded as a composite reflection of multiple developments: enhanced clinical care, improved diagnostic and death certification practices, and methodological refinements in data synthesis. Consequently, the observed reductions in ASMR and ASDR might not fully capture the true disease burden, particularly in China, where these metrics must be interpreted within the context of a population exceeding 1.4 billion.

Regarding sex-specific patterns, both the incidence and prevalence rate of IBD had been marginally higher among females than males since the early twenty-first century. However, the increasing trend had been more pronounced in males. Several mechanisms may underlie these sex-specific disparities. First, biological differences in encompassing hormonal fluctuations, genetic polymorphisms and organ-specific physiology modulated the way of drug metabolism and immunological response. Females generally exhibited more robust cellular and humoral immune responses than males, conferring a higher susceptibility to autoimmune disorders, including IBD (39). Second, estrogen exerted protective effects by attenuating systemic inflammation and limiting inflammatory cell infiltration and histological injury, thereby potentially retarding IBD progression (40, 41) and estradiol treatment significantly increased the colon length (42). Third, smoking was a well-established risk factor for IBD. According to the Global Adult Tobacco Survey, the prevalence of smoking was markedly higher among males (52.9%) than females (1.2%) in China (43). Males often exhibited dietary patterns characterized by higher consumption of fats, proteins, and sugar foods, along with relatively lower intakes of vegetables and fruits. Such dietary habits may disrupt the balance of intestinal microbiota, which played a crucial role in maintaining gut homeostasis (44).

Although the ASMR and ASDR among populations aged 0–54 years declined, males consistently exhibited substantially higher burdens of both death and disability. Elevated levels of androgens in males may exacerbate intestinal inflammation and drive the progression of IBD, leading to more severe disease presentations and an elevated risk of mortality (45). Moreover, excessive alcohol consumption can cause damage to the intestinal mucosa, induce or worsen intestinal inflammation, and further increase the disease burden among male patients with IBD (46). Males generally exhibited lower levels of health awareness of IBD compared to females. They tended to have reduced sensitivity to bodily discomfort and a lower propensity to seek timely medical treatment when symptoms arised (47). This behavior might delay the early diagnosis and treatment of IBD, thereby missing the optimal therapeutic window. Delayed diagnosis and treatment were associated with more severe disease progression and increased mortality rates in IBD, significantly amplifying the disease burden in affected patients. In Chinese society, males typically born a greater burden of economic and social responsibilities, resulting in higher levels of work and life related stress, which can exacerbate intestinal inflammation in IBD patients, potentially leading to more severe disease manifestations and complications. Additionally, psychological stress can negatively impact treatment adherence and effectiveness. This suboptimal engagement with medical interventions can result in inadequate disease control, increased risk of disease progression and complications, and ultimately higher mortality and DALYs among males with IBD.

When examining the age-related patterns, the incidence and prevalence rates of IBD exhibited an upward trend across multiple age groups. Notably, the most pronounced increases were observed in populations aged 45–49 years, likely due to a complex interplay of factors, including immune system dysfunction and decline, as well as the cumulative burden of intestinal diseases. With advancing age, changes in immune function may impair the regulation of inflammation, thereby contributing to the increased disease burden in this age group (48). This age-related decline in immune function, known as immunosenescence, may lead to increased susceptibility to chronic inflammatory conditions, including IBD. Moreover, the cumulative effects of chronic intestinal conditions, such as recurrent infections or dysbiosis, might predispose individuals to IBD by disrupting the intestinal mucosal barrier and altering the gut microbiota. These age-associated biological changes, together with lifestyle and environmental exposures, likely acted synergistically to elevate the risk of IBD in this age group (49).

Regard to the mortality rate and DALYs, a downward trajectory was observed across various age groups, with the most notable decline in age group 0–4 years. The rapid reduction in mortality and DALYs among children aged 0–4 years may be attributed to several factors. First, improved early diagnosis and treatment technologies enabled earlier detection of the disease and timely intervention. New drugs and treatment regimens continued to emerge, significantly improving the therapeutic effect of IBD. Second, widespread vaccination programs had effectively reduced the incidence of infectious diseases in children, indirectly lowering the risk of developing IBD. Additionally, improved nutritional status among children had enhanced immune system function and further reduced the risk of IBD (50). For example, the promotion of breastfeeding and the implementation of child nutrition supplementation programs had significantly improved children’s intestinal health. Third, with socioeconomic development, the allocation of medical resources had become more reasonable, allowing children to access more timely and high-quality medical services. Meanwhile, increased attention to children’s health from parents and medical professionals had facilitated the early detection and management of chronic diseases such as IBD. Finally, shifts in the spectrum of childhood diseases also contributed to the decline. As the incidence of infectious diseases in childhood had decreased, the relative burden of chronic non-infectious diseases like IBD has declined. These changes reflected the success of public health interventions (11, 51).

The age effect consistently suggested that both the incidence and prevalence of IBD increased with advancing age, aligning with previous age-stratified observations. This age-related rise in incidence and prevalence was likely attributable to multiple interrelated factors, encompassing alterations in immune system function, intestinal flora imbalance, lifestyle, environmental influences, dietary patterns, socioeconomic status, healthcare access, shifts in disease spectrum, diagnostic advancements, and the cumulative impact of environmental exposures. These interrelated factors highlighted the crucial need for early health education and the promotion of healthy lifestyles, and the implementation of targeted interventions to mitigate IBD-related risk factors. Proactive measures in these domains held the potential not only to reduce the incidence and prevalence of IBD but also improve the overall quality of life for affected individuals (52). The effect of age on mortality and DALYs exhibited an inverse pattern compared to that observed for incidence and prevalence. As age increased, both mortality rate and DALYs tended to decline. This trend was particularly pronounced in the 0–4 age group, highlighting the significance of early intervention and timely treatment for IBD.

With regard to the period effect, the incidence and prevalence of IBD in China suggested an upward trend from the 1990s until 2016, after which they began to decline. This trend was likely attributable to a combination of factors, including rapid economic growth, shifts in lifestyle, such as the adoption of a more Western-style diet and improvements in environmental hygiene, which may have elevated the risk of IBD. Concurrently, the increased availability of medical resources, advancements in diagnostic technologies, and improved awareness of IBD among healthcare professionals had facilitated more accurate diagnosis. In China, IBD was first recognized in 1956, and since then, consensus opinions on the diagnosis and treatment of IBD had been formulated in 1978, 1993, 2001, 2007, and 2012 (53). These consensus guidelines had played a significant role in standardizing and improving the clinical diagnosis and treatment of IBD in China. In 2016, the Chinese government launched the Healthy China Strategy, which aimed to enhance population health and promote health equity through strategic initiatives, such as encouraging healthy lifestyles, optimizing health services, and improving health security.

In terms of IBD prevention and treatment, the strategy had contributed to reducing the incidence and prevalence rates through measures, such as strengthening early screening and diagnosis, enhancing IBD management, advocating for healthy lifestyles, intensifying health education and public awareness campaigns. These multifaceted approaches had mobilized broad societal participation in the fight against IBD. The period effect suggested a temporal trend from 1992 to 2021, during which, both the mortality rate and the risk of DALYs declined, corroborating the findings of previous Joinpoint analyses. However, a notable gender-based difference emerged in prevalence, mortality, and DALYs. Specifically, since the early 21st century, the period effect was associated with a higher RR of prevalence in females compared to males. In contrast, for mortality and DALYs, the period effect conferred a greater RR to males than to females. These findings underscored the necessity of strengthening tertiary prevention strategies for males. it was imperative to implement targeted measures to prevent disease progression, reduce complications, and enhance both quality of life and survival rates in patients with IBD.

The cohort effect suggested a monotonic increase in both incidence and prevalence of IBD among successively later-born Chinese cohorts. In this study, the 1977–1981 birth cohort was used as the reference, and a stepwise increase in IBD risk was observed in subsequent cohorts. Prior to 1978, China experienced major events such as warfare, the founding of the People’s Republic of China, famines, and the cultural revolution. During which, living conditions were poor and the risk of developing and suffering from IBD remained relatively low. However, following the reform and opening-up, rapid industrialization, expanded international cooperation, and the adoption of Western dietary patterns coincided with markedly elevated IBD incidence and prevalence. In terms of gender, following the reform and opening-up, the risk of developing and suffering from IBD in females was higher than that in males, which was consistent with previous analysis.

In terms of mortality and DALYs, the risk gradually decreased across successive birth cohorts, which may be associated with the advancement in strategies for disease prevention, treatment, control, and management strategies. However, an interesting gender-related reversal was observed. Before the 1980s, females exhibited higher mortality risk from IBD than males; thereafter, the mortality risk in males exceeded that in females, potentially due to the social status of females. During times of turmoil, females often faced limited access to adequate nutrition necessary for maintaining physical health (54). However, it cannot be conclusively determined that the increase in mortality was directly caused by IBD, as it involved the combined influence of numerous social, economic, and cultural factors. Following the reform and opening-up, the mortality risk of IBD in males increased, potentially due to unhealthy lifestyles such as smoking, and alcohol consumption. Additionally, gender differences existed in IBD awareness, willingness to undergo screening, treatment adherence, and the social pressure may contribute to the higher mortality risk observed in males compared to females.

As described in the methods section, the results of the age-period-cohort model depended on the statistical constraints applied to address the inherent identifiability problem. Although we employed the relatively robust intrinsic estimator method—suggesting that the core trends are stable. This did not imply that the estimated effects represent absolute “true values.” The estimated period effects (capturing all period-specific influences, such as diagnostic capabilities and environmental changes) and cohort effects (reflecting early-life exposures) were intrinsically correlated. For example, the observed increase in risk among more recent birth cohorts may partly reflect that individuals in these cohorts underwent more frequent and sensitive examinations at younger ages—i.e., during more recent periods. Therefore, APC results were best interpreted as revealing the multidimensional structural patterns of disease burden over time and as providing strongly suggestive evidence for etiological hypotheses (e.g., the importance of early-life exposures), rather than as definitive causal conclusions.

The predictive model indicates that, among Chinese populations aged 0–54 years, the absolute number of incident IBD cases will decline through 2030, whereas the ASIR will continue to rise, with males consistently showing higher rates than females. This divergence highlights an emerging epidemiological transition and identifies a refined target for primary prevention strategies. The persistent increase in ASIR suggests that the relative risk among young and middle-aged adults is rising. Consequently, evidence-based interventions, such as promoting prudent dietary patterns and favorable lifestyle behavior, should be prioritized in this demographic. Although the projected number of prevalent cases is expected to stabilize, the ASIR among females is forecasted to decline, while male rates remain relatively constant. This sex disparity highlights the persistent challenge of engaging males in health education initiatives and risk factor modification program. Mortality and DALYs attributable to IBD are projected to decrease steadily. These favorable trends, driven by improved therapeutic algorithms and broader access to heathcare, shift the focus of future research toward optimizing quality of life metrics and minimizing both years of life lost and years lived with disability among prevalent patients with IBD.

From a methodological perspective, this projection may partly reflect an “over-extrapolation” effect arising when the ARIMA model fits the recently rapid declining trend. Accordingly, the principal value of the “near-zero mortality” prediction lies not in providing a precise numerical estimate, but in highlighting an important public health possibility: the theoretical feasibility of reducing IBD mortality to a negligible level. This projection establishes an ambitious yet informative quantitative benchmark for assessing the effectiveness of future prevention and treatment strategies. Achieving this goal—rather than treating it merely as a statistical extrapolation, requires sustained commitment, including ensuring comprehensive and equitable access to high-quality diagnosis and care, as well as pursuing critical advances in understanding pathological mechanisms and developing therapeutic strategies for refractory cases.

The ARIMA model relies on fitting and extrapolating historical data, operating under the assumption that, in the absence of major public health interventions, shifts in diagnostic or therapeutic paradigms, or external shocks, the observed trends in disease burden will continue into the future. This projection provides valuable insights for public health planning. Specifically, the model predicts a continued rise in incidence alongside a relatively stable prevalence, suggesting a growing cumulative patient population and increasing pressure on the healthcare system. These findings offer urgent, data-driven support for formally prioritizing IBD within the national chronic disease prevention and control framework.

Furthermore, the projections underscore a growing demand for specialized resources, including gastroenterologists, endoscopy centers, IBD-specialized nurses, and advanced therapeutics such as biologic agents. Health authorities can use these forecasts to proactively plan specialist training, allocate medical equipment, and budget for drug reimbursements, thereby mitigating potential service delivery shortages. These predicted trends can also inform specific, measurable public health targets. Moving forward, by comparing observed data with the baseline forecasts established in this study, it will be possible to directly assess the real-world effectiveness of interventions—such as early screening programs, standardized treatment protocols, and patient management initiatives—in altering the natural trajectory of the disease.

Our predictive model offers a data-driven baseline scenario to inform future planning of the IBD burden. However, it is essential to acknowledge that public health and environmental interventions, such as dietary modification and judicious antibiotic use may attenuate the rising incidence at its source. Concurrently, nationwide advances in endoscopic technology and heightened disease awareness are expected to promote earlier diagnosis and more standardized treatment, thereby modifying the natural disease course and slowing the progression from incidence to prevalence and disability burden. Moreover, the widespread adoption of novel, more effective, and more accessible therapies (e.g., targeted biologics, small-molecule agents, and stem cell therapies), together with improved healthcare accessibility and a strengthened medical security system, is likely to enhance overall disease management and reduce the total disease burden. In contrast, persistent socioeconomic and health shocks, such as pandemics similar to COVID-19, other socioeconomic disruptions, rapid urbanization, dietary Westernization, and increasing stress levels may further accelerate IBD onset, particularly CD, potentially driving incidence beyond historically projected trajectories.

Although this study analyzed disease burden of IBD in China, it is important to acknowledge the potential heterogeneity in epidemiological characteristics and disease burden between UC and CD. Previous machine learning based analyses had stratified the global progression of IBD into three distinct stages, with China currently classified as being in Stage 2 (CD incidence: 1.0–4.4 per 100,000 population; UC incidence: 2.3–6.3 per 100,000 population), whereas North America and Western Europe have already advanced to Stage 3(CD incidence: 6.6–14.0/10per 100,000 population, UC incidence: 10.1–18.1per 100,000 population) (55). China may transition to Stage 3 in the future, underscoring the need for proactive planning of healthcare resources. Previous studies have shown that, at the global level, the incidence of UC generally exceeds that of CD, with an approximate ratio of 2:1 (56). However, in China, the incidence ratio of UC to CD was markedly higher, reaching 12.61. Furthermore, the incidence rates of both UC and CD were higher among males than among females in the Chinese population (57). This discrepancy may reflect higher rates of delayed diagnosis or underdiagnosis of CD in China, or a greater genetic and/or environmental susceptibility to UC. Moreover, the incidence of both UC and CD in China demonstrated a clear regional gradient, with higher rates observed in southern provinces and more economically developed areas (e.g., Guangdong) than in northern regions (18). These patterns were likely associated with environmental factors related to urbanization and industrialization, such as dietary Westernization and antibiotic use, which is consistent with the inferences drawn in our study. Based on existing IBD research in China, strengthening national IBD surveillance, monitoring shifts in risk factors, and enhancing diagnostic capacity for CD (e.g., through endoscopic and imaging technologies) are recommended to reduce underdiagnosis. Future studies should further investigate the genetic underpinnings of the disproportionate UC/CD ratio in China—such as the relatively low mutation frequency of the NOD2 gene—as well as specific environmental influences, including the potentially protective role of high *Helicobacter pylori* infection prevalence against CD (19).

To our knowledge, this is the first study to systematically quantify and forecast the burden of IBD among Chinese populations aged 0–54 years by using the GBD2021 database. The findings provide valuable evidence to inform the formulation of prevention and control strategies for government policymakers. Meanwhile, we systematically analyzed the changing trends in disease burden across males or females and age, which is crucial for understanding the epidemiological characteristics of IBD in China. Nevertheless, our study still has certain limitations. First, a primary limitation of this study arises from its reliance on modeled estimates generated by GBD 2021. As a result, the nationally aggregated trends reported here (e.g., national ASIR) may not fully reflect the specific epidemiological profiles of high or low burden provinces, and the upward trend in IBD incidence is likely steeper than the national average in developed eastern coastal regions, characterized by high urbanization and increasingly “Westernized” dietary patterns. Limiting the generalizability of our findings to particular subpopulations. Second, the GBD estimates are ultimately calibrated to routinely reported data, which may systematically underestimate the incidence of IBD, particularly Crohn’s disease—in regions with weaker healthcare infrastructure, where endoscopic capacity is limited, referral pathways are incomplete, and diagnostic coding is inconsistent. Third, due to the constraints of the GBD 2021 database, this study could not conduct independent trend analyses for the two major subtypes of IBD, UC and CD. The lack of subtype-specific data limits the ability to examine shifts in the disease spectrum in detail and to assess subtype-specific therapeutic needs. Fourth, this study is primarily a descriptive epidemiological analysis, aimed at characterizing temporal trends and forecasting the future burden of IBD within a defined population. Although the observed rise in incidence aligns with factors such as the adoption of a more “Westernized” lifestyle and widespread improvements in diagnostic capacity reported in the literature, our analysis did not incorporate specific proxy indicators for these risk factors or for healthcare accessibility. Fifth, the ARIMA model employed in this study is an extrapolative forecasting tool based on historical time-series data. It does not account for potential future disruptions or innovations, which could substantially alter the trajectory of IBD burden.

Nevertheless, analyses based on GBD data remain invaluable. They provide the most systematic and comparable quantitative framework currently available for understanding the long-term, large-scale evolution of IBD in China and its burden structure, including trends in mortality and DALYs through secondary analyses, such as Joinpoint regression to detect turning points, age-period-cohort model for trend decomposition, and forecasting based on national trends. Our study has revealed important nuances within this overall trajectory, including sex disparities and shifting age patterns. Looking forward, it is essential to establish a high-quality national IBD registry in China, conduct population-based epidemiological surveys, and implement large-scale prospective cohort studies to identify specific environmental and genetic risk factors. Such initiatives will be critical for calibrating model estimates, uncovering regional heterogeneity, and ultimately informing more targeted prevention and control strategies.

## Conclusion

The increasing ASPR among populations aged 0–54 years in China results from combinations of declining ASMR and ASDR, alongside a rising ASIR between 1992 and 2021 in China. The growing incidence warrants serious attention. IBD is more prevalent in females compared to males. The risk of incidence and prevalence rose with increasing age, and is higher in females. The risk of developing and living with IBD has declined in recent years. Meanwhile, mortality and DALYs continue to decrease across age, period and cohort, although mortality and DALYs remain higher in males in recent cohorts. Given the large population and increasing trend among young and middle-aged adults, multiple interventions should be considered to minimize the burden of IBD in the future.

## Data Availability

The datasets presented in this study can be found in online repositories. The names of the repository/repositories and accession number(s) can be found at: The primary data source is the GBD 2021 Results Tool, accessible at https://vizhub.healthdata.org/gbd-results/.

## Footnotes

1
https://analysistools.cancer.gov/apc/

2
https://analysistools.cancer.gov/apc/
