# Supplementary figures and images for: The temporal trends of incidence, prevalence, mortality, and disability-adjusted life years of inflammatory bowel disease in the Chinese population aged 0–54 years and prediction to 2030
Source: Front Med (Lausanne). 2026 Feb 25;13:1689227. doi: 10.3389/fmed.2026.1689227 (PMC12975574; doi:10.3389/fmed.2026.1689227)

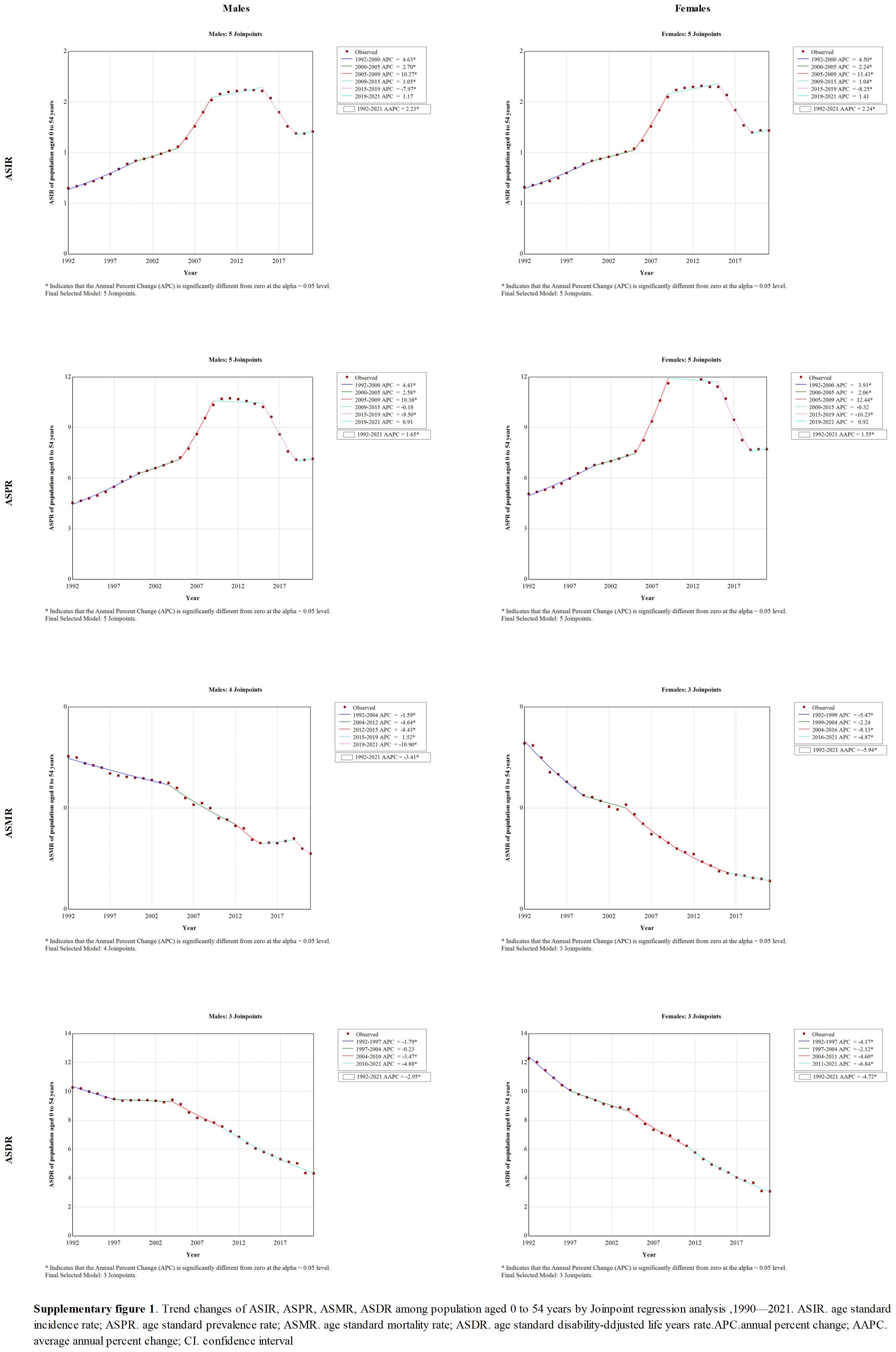

Supplement: Supplementary file 2 [file Image_1.jpeg]
